# Yeast Two-Hybrid Systems and Protein Interaction Mapping Projects for Yeast and Worm

**DOI:** 10.1002/1097-0061(20000630)17:2<88::AID-YEA20>3.0.CO;2-Y

**Published:** 2000

**Authors:** Albertha J. M. Walhout, Simon J. Boulton, Marc Vidal

**Affiliations:** Dana-Farber Cancer Institute and Department of Genetics Harvard Medical School 44 Binney Street Boston MA 02115 USA

## Abstract

The availability of complete genome sequences necessitates the development of standardized functional assays to analyse the tens of thousands of predicted gene products in high-throughput experimental settings. Such approaches are collectively referred to as ‘functional genomics’. One approach to investigate the properties of a proteome of interest is by systematic analysis of protein–protein interactions. So far, the yeast two-hybrid system is the most commonly used method for large-scale, high-throughput identification of potential protein–protein interactions. Here, we discuss several technical features of variants of the two-hybrid systems in light of data recently obtained from different protein interaction mapping projects for the budding yeast *Saccharomyces cerevisiae* and the nematode *Caenorhabditis elegans*.


**Yeast**
*Yeast* 2000; **17**: 88–94.

**Review Article**

### Yeast two-hybrid systems and protein interaction mapping projects for yeast and worm

Albertha J. M. Walhout, Simon J. Boulton and Marc Vidal*
*Dana-Farber Cancer Institute and Department of Genetics, Harvard Medical School, 44 Binney Street, Boston, MA 02115, USA*

**Correspondence to: M. Vidal, Dana-Farber Cancer Institute and Department of Genetics, Harvard Medical School, 44 Binney Street, Boston, MA 02115, USA.*

### Abstract

**The availability of complete genome sequences necessitates the development of standardized functional assays to analyse the tens of thousands of predicted gene products in high-throughput experimental settings. Such approaches are collectively referred to as 'functional genomics'. One approach to investigate the properties of a proteome of interest is by systematic analysis of protein–protein interactions. So far, the yeast two-hybrid system is the most commonly used method for large-scale, high-throughput identification of potential protein–protein interactions. Here, we discuss several technical features of variants of the two-hybrid systems in light of data recently obtained from different protein interaction mapping projects for the budding yeast *Saccharomyces cerevisiae* and the nematode *Caenorhabditis elegans*. Copyright © 2000 John Wiley & Sons, Ltd.**

**Keywords: protein interaction mapping; yeast two-hybrid; co-immunoprecipitation; GST pull-down; two-hybrid matrix approach**

### Introduction

Several (near) complete genome sequences of model organisms have recently been released. Annotation of these genomes has led to the prediction of ~4000 protein-encoding open reading frames (ORFs) for *Escherichia coli*, ~6000 ORFs for the yeast *S. cerevisiae*, ~13 000 ORFs for *Drosophila melanogaster* and ~19 000 for *C. elegans* [1,3,7,13]. The human genome sequence is anticipated this year and is expected to lead to the prediction of more than 100 000 ORFs [6,26]. By themselves, such predicted ORF sequences offer little information about the function of their predicted protein products.

Comparative genomics can be used to annotate the function of large numbers of predicted and previously uncharacterized gene products. For example, a comparison between the complete set of predicted ORFs in *S. cerevisiae* and *C. elegans* resulted in the definition of orthologues for 2497 yeast ORFs and 3653 worm ORFs, respectively ($E < 10^{-10}$) [4]. In addition, half of the predicted fly proteins show significant homology to mammalian proteins ($E < 10^{-10}$) [22]. However, comparative genomics is not applicable to the many predicted gene products for which no recognizable conserved domains can be detected using current BLAST methods.

In order to accelerate functional annotations for such predicted gene products, several functional genomics projects need to be initiated. Such projects utilize standardized functional assays to analyse large sets of genes/proteins simultaneously. One of the most established functional genomics approaches that has been used so far is expression-profiling using DNA chips or microarrays [19,23]. For example, this technique has been used to annotate genes in yeast whose transcription changes during sporulation [5]. Even though expression-profiling experiments yield a wealth of information on gene expression, they offer little annotation for the protein complement, or proteome, of an organism. Hence, large-scale analyses of gene products (protein) are required to add a level of complexity to data obtained from gene (DNA) analyses. Ultimately, data obtained from different functional genomics strategies should be integrated to allow the formulation of meaningful hypotheses [28; Walhout and Vidal, Protein interaction maps for model organisms. *Trends Biochem Sci*, in preparation].

Copyright © 2000 John Wiley & Sons, Ltd.

### Interaction-detection techniques

Most proteins require physical interactions with other proteins to fulfil their biological role. Therefore, it has been proposed that functional annotations for proteomes can be obtained by systematically identifying potential protein–protein interactions [18]. Several commonly used protein–protein detection techniques have been described, including co-immunoprecipitation, gluthatione-*s*-transferase (GST) pull-down experiments and yeast two-hybrid analyses (Figure 1). By co-immunoprecipitation (Figure 1A), endogenously interacting proteins can be purified using specific antibodies [14]. Protein–protein interactions observed using this method do not necessarily have to be direct but are deemed relevant interactions *in vivo*. For example, protein X may indirectly associate with Z through a bridging protein Y (Figure 1A). Although interaction data obtained from co-immunoprecipitation experiments are likely to be biologically relevant, it is not yet feasible to perform such experiments on a proteome-wide scale, since it remains technically challenging to generate antibodies for each predicted protein. For GST pull-down experiments, proteins are exogenously expressed as GST-fusion proteins (GST-X) and purified on glutathione-agarose (GA) beads (Figure 1B) [15]. In general, purified GST-X is subsequently incubated with cellular extracts and

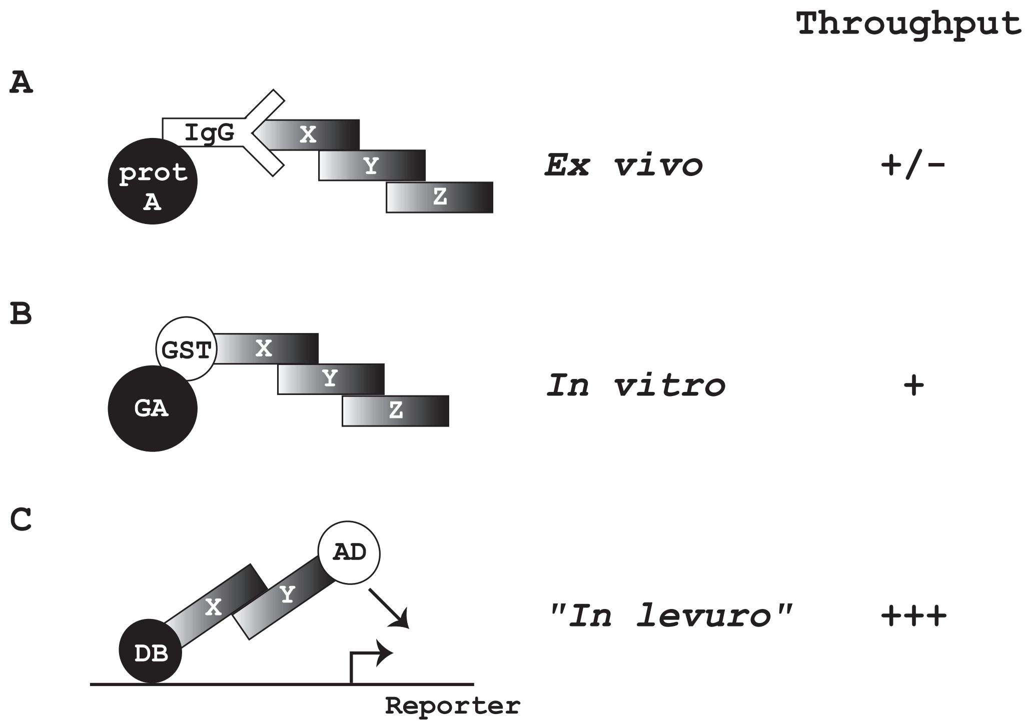

**Figure 1.** Non-exhaustive list of techniques commonly used to detect protein–protein interactions. Several techniques have been described for the detection of protein–protein interactions. (A) Co-immunoprecipitations using specific antibodies (IgG) allow the purification of endogenous proteins with protein A (prot A) coupled beads. (B) glutathione-*s*-transferase (GST) fusion proteins can be expressed in bacteria or other expression systems, purified and used to 'pull down' interaction partners on glutathione–agarose (GA) beads. (C) With the yeast two-hybrid system, protein–protein interactions can be detected in living yeast cells ('*in levuro*', Figure 1C). Protein X is fused to the DNA binding domain (DB) of a transcription factor and the potential interaction partners are fused to a transcription activation domain (AD-Y). Upon an interaction between X and Y, a functional transcription factor is reconstituted that can activate the expression of specific reporter genes. In both co-immuno-precipitations and GST pull-down assays, multiple proteins can be precipitated that do not necessarily have to interact directly with the bait protein X. Here, an example is shown in which protein Y binds directly to X. Protein Z is precipitated with anti-X antibodies or GST-X via its interaction with protein Y. Theoretically, bridging proteins could also facilitate interactions in the context of the yeast two-hybrid system. However, in this case, the identity of such proteins remains elusive

Copyright © 2000 John Wiley & Sons, Ltd.

complexes are purified using GA beads. Both biochemical approaches make use of protein separation by gel electrophoresis and determination of associated protein identity by mass spectrometry [20,24]. Recently, the (near) complete yeast proteome was fused to GST and used in a biochemical approach, in which proteins were annotated by their associated enzymatic activities [21]. However, such a set of GST-X fusion protein has not yet been used for large-scale protein interaction mapping.

In the yeast two-hybrid system [8] (Figure 1C), a protein of interest, X, is fused to the DNA binding domain (DB) of a transcription factor, such as Gal4p. The second hybrid protein, Y, is fused to a transcriptional activation domain (AD). A physical interaction between X and Y results in the reconstitution of a functional transcription factor that can activate expression of reporter genes. Usually, reporter genes that allow growth selection on specific media are used. The two-hybrid system is carried out *in vivo* and only requires the manipulation of DNA. As a consequence, the two-hybrid system is more amenable to automation and can be used to analyse large sets of (predicted) proteins simultaneously. Recently, the yeast two-hybrid system has been used to initiate the generation of protein interaction maps for *S. cerevisiae* and *C. elegans* [16,25,29].

### False positives and false negatives

One intrinsic caveat of the yeast two-hybrid system is the potential detection of spurious interactions that bear no biological significance. The occurrence of such false positives can be reduced using low expression levels of the two hybrid proteins and the use of multiple reporter genes utilizing different promoters [27]. As a consequence of the artificial nature of the two-hybrid system, interactions should be viewed as hypotheses until they are validated in the appropriate biological system. A number of proteins are frequently detected using multiple baits and might behave notoriously as false positives. Although these proteins are likely to interact with other proteins to fulfil their biological role themselves, they should be treated with caution when found in any two-hybrid experiment.

In contrast to the detection of false positives, a number of reported interactions can not be readily detected in the two-hybrid system and are therefore deemed false negatives. False negatives can be caused by different characteristics of the two-hybrid system. First, DB-X and/or AD-Y may fail to localize to the yeast nucleus. Second, X and/or Y may be unable to function within the context of a DB or AD fusion. Third, the interaction between X and Y may depend on post-translational modifications that are absent in yeast cells. Finally, it has been reported that a number of protein–protein interactions can only be detected in the two-hybrid system when either X or Y is truncated. Recently, we have estimated the percentage of false negatives in our two-hybrid system to be approximately 45% [29]. This suggests that large-scale two-hybrid analyses are useful to obtain partial coverage of protein–protein interactions within a proteome of interest. However, in order to attain (near) complete protein interaction contiguity, alternative large-scale approaches will have to be developed.

### Two-hybrid variants

The two-hybrid system was initially developed to test known interactions between two proteins [8] (Figure 1C). Subsequently, it was applied as a method for the identification of novel potential protein–protein interactions, using cDNA libraries fused to AD (AD–cDNA) (Figure 2A). Often a single full-length DB-X bait protein of interest is used. Potential interaction partners obtained are frequently retrieved as fragments, since AD–cDNA fusion libraries are generated by Reverse Transcription and therefore do not exclusively contain full-length ORFs. When working with model organisms for which a complete genome sequence is available, a single sequence tagging reaction is sufficient to identify the potential interactor. Thus we refer to potential interactions as 'interaction sequence tags' or 'ISTs'. The AD–cDNA approach has the advantage of partially defining the region of protein AD-Y required for the interaction. Indeed, in many cases independently derived clones of the same ORF differ in length but share a common region required for the interaction. The detection of multiple overlapping fragments has been proposed as a criterion for the classification of interaction data [12]. This approach has been utilized on a larger scale for 27 *C. elegans* proteins involved in vulval development and resulted in the identification of 148 worm ISTs (Table 1) [29]. In addition, it

Copyright © 2000 John Wiley & Sons, Ltd. 

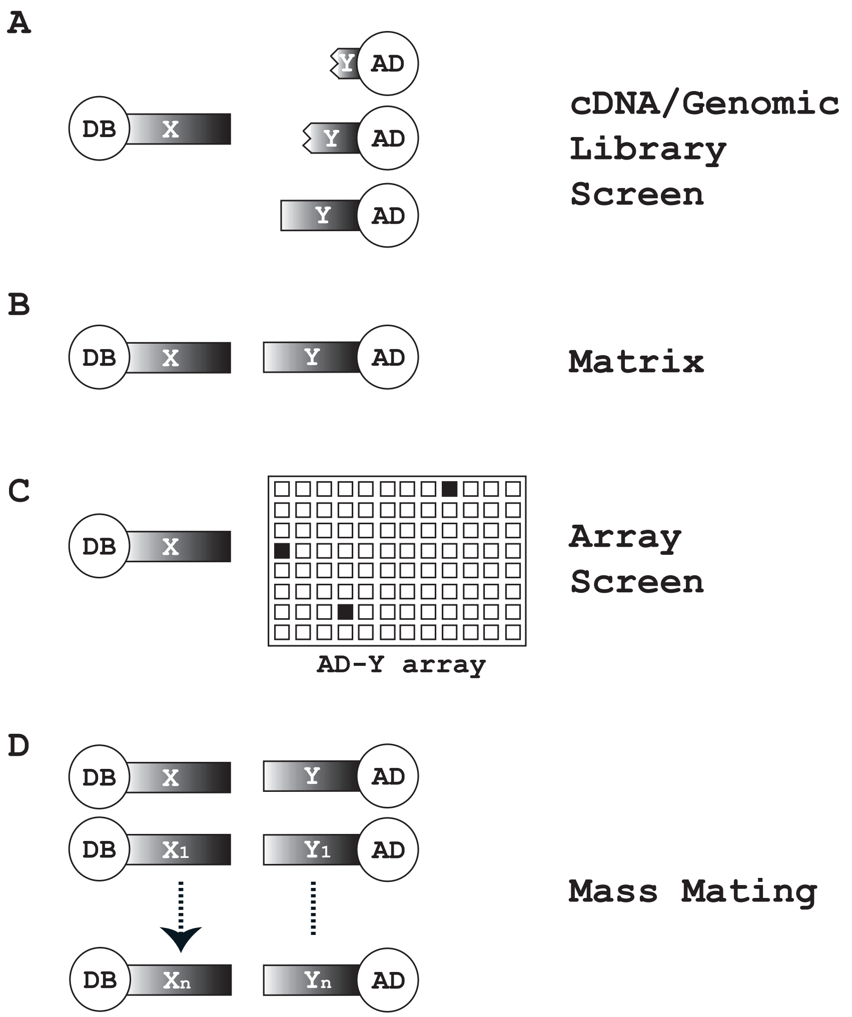

**Figure 2.** Two-hybrid strategies. (A) In 'classical' two-hybrid screens, a single protein of interest is fused to DB and used to screen for potential interaction partners in an AD-Y cDNA library. (B) In a two-hybrid matrix experiment, proteins of interest are fused both to DB and to AD and expressed in yeast of opposite mating types. Subsequently, each pair-wise DB-X/AD-Y combination is generated by mating and tested for two-hybrid interaction phenotypes. Usually, the sequence of both X and Y has been determined prior to the two-hybrid experiment, circumventing the need for sequencing analysis. (C) The two-hybrid array approach resembles the matrix approach. However, large or complete sets of AD-Y expressing yeast strains are arrayed onto 96- or 384-well plates and mated with a yeast strain of the opposite mating type that expresses a single DB-X protein. Black squares indicate potential positives in which an interaction results in a selectable phenotype. (D) In the 'mass mating' approach, sets of DB-X proteins are pooled and mated with pools of AD-Y expressing yeast cells. ISTs are identified by plating diploids onto selective media, followed by sequencing of both the DB and AD inserts.

has been used for yeast proteins involved in splicing, which resulted in the identification of 170 ISTs and the related Lsm proteins that resulted in 263 ISTs (Table 1) [12].

An alternative two-hybrid 'matrix' approach has recently been employed [29]. A matrix experiment utilizes large sets of full-length ORFs, often functionally related, that are each fused to DB and AD. Usually, DB-X and AD-Y are transformed into yeast of opposite mating types (*MAT***a** and *MAT*$\alpha$ respectively). Each pair-wise combination of DB-X and AD-Y is generated by mating and examined for two-hybrid interaction phenotypes (Figure 2B). This approach was initially used to identify protein–protein interactions between *Drosophila* proteins involved in cell cycle regulation [9].

Copyright © 2000 John Wiley & Sons, Ltd.

Table 1. Comparison of IST data obtained from different protein interaction mapping projects

| # DB-ORFs | # ISTs | ISTs/ORF | Organism | Approach | Report |
|---|---|---|---|---|---|
| 11 | 263 | 23.9 | *S. cerevisiae* | 2A/2B | Fromont-Racine *et al.* 2000 [11] |
| 15 | 170 | 11.3 | *S. cerevisiae* | 2A | Fromont-Racine *et al.* 1997 [12] |
| 16 | N/A | N/A | *S. cerevisiae* | 2A | Flores *et al.* 1999 [10] |
| 29 | 156 | 5.4 | *C. elegans* | 2A/2B | Walhout *et al.* 2000 [29] |
| 55 | 25 | 0.5 | T7 | 2B/2D | Bartel *et al.* 1996 [2] |
| 159 | 183 | 1.2 | *S. cerevisiae* | 2D | Ito *et al.* 2000 [14] |
| 192 | 281 | 1.5 | *S. cerevisiae* | 2C | Uetz *et al.* 2000 [25] |
| 5345 | 692 | 0.1 | *S. cerevisiae* | 2D | Uetz *et al.* 2000 [25] |

#DB-ORFs: the number of different baits used. #ISTs: the number of different ISTs found. Each IST represents a single (predicted) ORF. ISTs/ORF: the number of ISTs found on average per ORF. Yeast two-hybrid approach used (refer to Figure 2: 2A = cDNA library screen; 2B = matrix screen; 2C = array screen; 2D = mass mating).

Matrix experiments provide the advantage of knowing the identity of each DB-X and AD-Y pair, thus circumventing the need for sequencing. However, this strategy provides no information about the domain of the protein that confers interaction.

In order to perform matrix experiments on a proteome-wide scale, arrays have been generated containing each predicted protein fused to AD (AD-Y) in a 384-well format (Figure 2C) [25]. ISTs are identified by mating a single DB-X bait with the complete AD-Y array. This approach has been used for 192 DB-X yeast proteins and resulted in 281 interacting pairs (Table 1) [25].

Another large-scale two-hybrid strategy that has been utilized recently is the mating of pools of DB-X and AD-Y fusion proteins and selection of potential interacting pairs on appropriate media (Figure 2D). In this approach, the identity of both X and Y has to be determined by sequencing. Two independent large-scale experiments using predicted yeast proteins resulted in 183 and 692 ISTs, respectively (Table 1) [16,25]. Data obtained from large-scale two-hybrid experiments are usually disseminated to the community via the Internet (**http://www.pnas.org**, **http://portal.curagen.com** and **http://www.vidal.dfci.harvard.edu**).

### Making sense of interaction data

In order to 'make sense' of the wealth of protein interaction data that has been released, several interaction classification strategies have been proposed. First, protein–protein interactions can be systematically compared between different model organisms. It is believed that conserved interactions, or 'interologs' (Figure 3A), have a higher likelihood of being biologically relevant. Several interologs have been described for interactions involving vulval proteins in *C. elegans* [29]. For example, a number of interactions in the Ras/Map-kinase pathway, previously reported between the mammalian proteins, were also found with the orthologous *C. elegans* proteins [29].

Second, analysis of protein interaction maps has led to the identification of interaction clusters. An interaction cluster can be viewed as a circular contig of protein interactions (Figure 3B). For example, X binds to Y, Y binds to Z, Z binds to W and W itself binds to X. Proteins found together within an interaction cluster may be part of a protein complex and therefore are more likely to function in a common process. For example, proteins comprising molecular machines, such as components of the RNA polymerase II holoenzyme [17], are likely to be found in a yeast two-hybrid interaction cluster.

### Where are we now?

The different protein interaction mapping projects published previously have generated relatively high numbers of ISTs. However, it is important to estimate the extent of coverage obtained from these different projects. It is widely accepted that most, if not all, proteins require interactions with other polypeptides to mediate their function. But, it has so far remained difficult to estimate the average number of interactors per protein in any proteome. The average of the number of ISTs found per bait for the different projects described here is approxi-

Copyright © 2000 John Wiley & Sons, Ltd. 

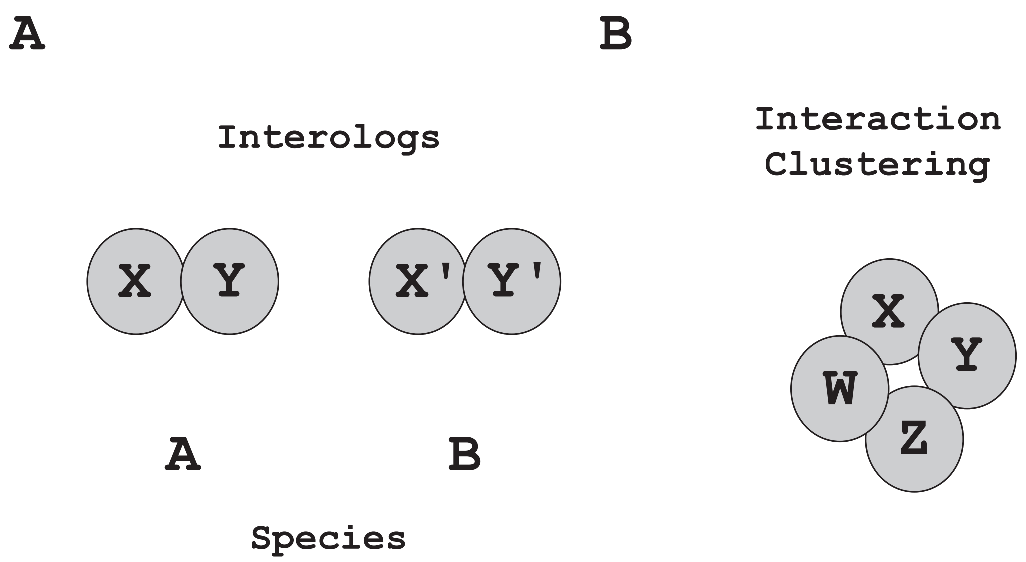

**Figure 3.** Classification of IST data. Over the next few years, hundreds of thousands of ISTs are anticipated for the different model organisms. ISTs merely represent hypotheses that should be validated in the appropriate biological system. Therefore, strategies for classification of ISTs have been developed to prioritize which ISTs are most likely to be biologically relevant. (A) When protein–protein interactions are conserved throughout evolution, i.e. they are detected in different model organisms, they are most likely to be biologically relevant. We refer to such interactions as 'interologs'. Using databases such as WormPD and YPD (**http://www.proteome.com**), ISTs can be compared between yeast and worms. (B) Interaction clustering provides a classification of ISTs that are more likely to be relevant *in vivo*. An IST cluster is identified as a contig of interactions. For example, when X binds to Y, which binds to Z, which binds to Y and if W binds to X, the four proteins form an IST cluster mately six (Table 1). This number could serve as a first approximation of the number of ISTs expected per bait in a proteome of interest. Therefore, an estimate of the number of expected ISTs for the yeast and worm proteomes could be as high as 120 000 and 36 000, respectively. So far, large-scale protein interaction mapping projects have generated ~150 ISTs for *C. elegans* (~0.13%) and ~1150 ISTs ( ~3%) for *S. cerevisiae*. In the relatively small-scale *C. elegans* project, 29 baits were used and larger-scale projects are underway (A. J. M. Walhout, S. J. Boulton and M. Vidal, unpublished data). For yeast, Ito *et al*. [16] have covered ~10% of yeast DB-X baits against the yeast predicted proteome and have found 182 ISTs. Similarly, Uetz *et al*. [25] performed an extensive screen of the yeast proteome and identified 957 putative interacting pairs. Although impressive, these numbers do not yet approximate to the expected number of yeast ISTs. Thus, it is possible that approximately ~0.6% and 3% of the yeast protein interaction maps are available from Ito *et al*. and Uetz *et al*., respectively. Consistent with this possibility, 28 of the 183 ISTs (15%) found by Ito *et al*. were also found by Uetz *et al*. Furthermore, the number of ISTs found per bait differs dramatically between the different projects (Table 1). Together, these observations indicate that the different two-hybrid strategies are complementary, but do not yield identical results. In addition, it shows that the screening has not been performed to saturation, emphasizing the need for more of these experiments.

### Conclusion

The data summarized above demonstrate that independent two-hybrid screens (Figure 2A) yield the largest numbers of ISTs. However, such screens are very time-consuming and elaborate. In contrast, the array (Figure 2C) and mass mating (Figure 2D) yeast two-hybrid strategies can be carried out at a higher throughput, but the rate of false negatives seems relatively high. Furthermore, it should be noted that even if all different two-hybrid

Copyright © 2000 John Wiley & Sons, Ltd.

approaches were performed until saturation was achieved, not every existing protein–protein interaction would be detected. Therefore, future protein interaction maps should comprise a compilation of data obtained from different large-scale projects, including two-hybrid screens.

### Acknowledgements

The work from this laboratory was supported by Grants 1 RO1 HG01715-01 (NHGRI) and 1 R21 CA81658 A 01 (NCI) awarded to M. V.

### References

1. Adams MD, *et al.* 2000. The genome sequence of *Drosophila melanogaster*. *Science* **287**: 2185–2195.
2. Bartel PL, Rocclein JA, SenGupta D, Fields S. 1996. A protein linkage map of *Escherichia coli* bacteriophage T7. *Nat Gen* **12**: 72–77.
3. Blattner FR, Plunkett GR, Bloch CA, Perna NT, Burland V, Riley M, Collado-Vides J, Glasner JD, Rode CK, Mayhew GF, Gregor J, Davis NW, Kirkpatrick HA, Goeden MA, Rose DJ, Mau B, Shao Y. 1997. The complete genome sequence of *Escherichia coli* K-12. *Science* **277**: 1453–1474.
4. Chervitz SA, Aravind L, Sherlock G, Ball CA, Koonin EV, Dwight SS, Harris MA, Dolinski K, Mohr S, Smith T, Weng S, Cherry JM, Botstein D. 1998. Comparison of the complete protein sets of worm and yeast: orthology and divergence. *Science* **282**: 2022–2027.
5. Chu S, DeRisi J, Eisen M, Mulholland J, Botstein D, Brown PO, Herskowitz I. 1998. The transcriptional program of sporulation in budding yeast. *Science* **282**: 699–705.
6. Collins FS, Patrinos A, Jordan E, Chakravarti A, Gesteland R, Walters L. 1998. New goals for the US Human Genome Project: 1998–2003. *Science* **282**: 682–689.
7. The *C. elegans* Sequencing Consortium. 1998. Genome sequence of the nematode *C. elegans*: a platform for investigating biology. *Science* **282**: 2012–2018.
8. Fields S, Song O. 1989. A novel genetic system to detect protein–protein interactions. *Nature* **340**: 245–246.
9. Finley RL Jr, Brent R. 1994. Interaction mating reveals binary and ternary connections between *Drosophila* cell cycle regulators. *Proc Natl Acad Sci U S A* **91**: 12980–12984.
10. Flores A, Briand JF, Gadal O, Andrau JC, Rubbi L, Van Mullem V, Buschiero C, Goussot M, Marck C, Carles C, Thuriaux P, Sentenac A, Werner M. 1999. A protein–protein interaction map of yeast RNA polymerase III. *Proc Natl Acad Sci U S A* **96**: 7815–7820.
11. Fromont-Racine M, Mayes AE, Brunet-Simon A, Rain J-C, Dix I, Joly N, Beggs JD, Legrain P. 2000. Genome-wide protein interaction screens reveal functional networks involving Sm-like proteins. *Comparative Functional Genomics* **17** (2): 95–110.
12. Fromont-Racine M, Rain JC, Legrain P. 1997. Toward a functional analysis of the yeast genome through exhaustive two-hybrid screens. *Nature Genet* **16**: 277–282.
13. Goffeau A, *et al.* 1997. The yeast genome directory. *Nature* **387** (Suppl.): 1–105.
14. Harlow E, Lane D. 1988. *Antibodies: A Laboratory Manual.* Cold Spring Harbor Laboratory Press: New York.
15. Harris M. 1998. Use of GST-fusion and related constructs for the identification of interacting proteins. *Methods Mol Biol* **88**: 87–99.
16. Ito T, Tashiro K, Muta S, Ozawa R, Chiba T, Nishizawa M, Yamamoto K, Kuhara S, Sakaki Y. 2000. Toward a protein–protein interaction map of the budding yeast: a comprehensive system to examine two-hybrid interactions in all possible combinations between yeast proteins. *Proc Natl Acad Sci U S A* **97**: 1143–1147.
17. Koleske AJ, Young RA. 1994. An RNA polymerase II holoenzyme responsive to activators. *Nature* **368**: 466–469.
18. Lander ES. 1996. The new genomics: global views of biology. *Science* **274**: 536–539.
19. Lockhart DJ, Dong H, Byrne MC, Follettie MT, Gallo MV, Chee MS, Mittmann M, Wang C, Kobayashi M, Horton H, Brown EL. 1996. Expression monitoring by hybridization to high-density oligonucleotide arrays. *Nature Biotechnol* **14**: 1675–1680.
20. Mann M, Wilm M. 1995. Electrospray mass spectrometry for protein characterization. *Trends Biochem Sci* **20**: 219–224.
21. Martzen MR, McCraith SM, Spinelli SL, Torres FM, Fields S, Grayhack EJ, Phizicky EM. 1999. A biochemical genomics approach for identifying genes by the activity of their products. *Science* **286**: 1153–1155.
22. Rubin GM, *et al.* 2000. Comparative genomics of the eukaryotes. *Science* **287**: 2204–2215.
23. Schena M, Shalon D, Davis RW, Brown PO. 1995. Quantitative monitoring of gene expression patterns with a complementary DNA microarray. *Science* **270**: 467–470.
24. Shevchenko A, Jensen ON, Podtelejnikov AV, Sagliocco F, Wilm M, Vorm O, Mortensen P, Shevchenko A, Boucherie H, Mann M. 1996. Linking genome and proteome by mass spectrometry: large-scale identification of yeast proteins from two dimensional gels. *Proc Natl Acad Sci U S A* **93**: 14440–14445.
25. Uetz P, Giot L, Cagney G, Mansfield TA, Judson RS, Knight JR, Lockshon D, Narayan V, Srinivasan M, Pochart P, Qureshi-Emili A, Li Y, Godwin B, Conover D, Kalbfleisch T, Vijayadamodar G, Yang M, Johnston M, Fields S, Rothberg JM. 2000. A comprehensive analysis of protein–protein interactions in *Saccharomyces cerevisiae*. *Nature* **403**: 623–627.
26. Venter JC, Adams MD, Sutton GG, Kerlavage AR, Smith HO, Hunkapiller M. 1998. Shotgun sequencing of the human genome. *Science* **280**: 1540–1542.
27. Vidal M, Legrain P. 1999. Yeast forward and reverse 'n'-hybrid systems. *Nucleic Acids Res* **27**: 919–929.
28. Walhout AJM, Endoh H, Thierry-Mieg N, Wong W, Vidal M. 1998. A model of elegance. *Am J Hum Genet* **63**: 955–961.
29. Walhout AJM, Sordella R, Lu X, Hartley JL, Temple GF, Brasch MA, Thierry-Mieg N, Vidal M. 2000. Protein interaction mapping in *C. elegans* using proteins involved in vulval development. *Science* **287**: 116–122.

Copyright © 2000 John Wiley & Sons, Ltd. 
